# High COVID-19 morbidity and mortality risk among smoked drug users in Brazil

**DOI:** 10.47626/2237-6089-2021-0290

**Published:** 2023-05-02

**Authors:** Vanessa Loss Volpatto, Ellen Mello Borgonhi, Felipe Ornell, Daniela Vicente Bavaresco, Helena Ferreira Moura, Francisco Diego Rabelo-da-Ponte, Felix Henrique Paim Kessler

**Affiliations:** 1 Centro de Pesquisa em Álcool e Drogas Hospital de Clínicas de Porto Alegre Universidade Federal do Rio Grande do Sul Porto Alegre RS Brazil Centro de Pesquisa em Álcool e Drogas, Hospital de Clínicas de Porto Alegre, Universidade Federal do Rio Grande do Sul (UFRGS), Porto Alegre, RS, Brazil.; 2 Programa de Pós-Graduação em Psiquiatria e Ciências do Comportamento UFRGS Porto Alegre RS Brazil Programa de Programa de Pós-Graduação em Psiquiatria e Ciências do Comportamento, UFRGS, Porto Alegre, RS, Brazil.; 3 Departamento de Clínica Médica Faculdade de Medicina Universidade de Brasília Brasília DF Brazil Departamento de Clínica Médica, Faculdade de Medicina, Universidade de Brasília, Brasília, DF, Brazil.; 4 Laboratório de Psiquiatria Molecular Hospital de Clínicas de Porto Alegre UFRGS Porto Alegre RS Brazil Laboratório de Psiquiatria Molecular, Hospital de Clínicas de Porto Alegre, UFRGS, Porto Alegre, RS, Brazil.

**Keywords:** COVID-19, smoke, crack/cocaine, tobacco, marijuana

## Abstract

In much of the West, including Brazil, drug use has increased since social distancing began in response to the pandemic. Use of smoked and modified drugs, and their impacts on health, may contribute to aggravate the effects of the pandemic. However, studies on the relationship between use of smoked drugs and the new coronavirus are still scarce and have not received enough attention in global health recommendations. This paper aims to briefly review the relationship between use of smoked drugs and acute respiratory syndrome coronavirus 2 [SARS-CoV-2]. Recent studies also suggest that drug consumption increases the risk of contamination by SARS-CoV-2 and leads to worse prognosis, particularly consumption of drugs that affect lung function. Use of smoked drugs, especially tobacco, is strongly associated with lung diseases that are risk factors for contamination by SARS-CoV-2. It is essential to develop strategies based on specific characteristics of drug users and for mental health professionals to be included in strategic teams. It is also necessary to invest in information campaigns regarding risks and prevention of harm caused by smoked drugs as well as to design strategies that facilitate access to psychosocial treatment during the pandemic.

Severe acute respiratory syndrome coronavirus 2 [SARS-Cov-2] is a virus that can cause respiratory diseases and other inflammatory illnesses, with poor outcomes. Since the beginning of the coronavirus pandemic, thousands of people have been infected worldwide.^1,2^ The mortality rate due to SARS-CoV-2 infection varies across locations (from 0.00% to 1.63%, corrected values from 0.00% to 1.54%)^3^ and may change substantially depending on health care infrastructure and prevention measures.^4,5^ There is an urgent need to identify risk factors for infection by SARS-CoV-2 and for progression to worse clinical outcomes. Although it is well documented that some diseases increase the risk of death from the Coronavirus disease 2019 (COVID-19), this relationship is not yet well understood. Recent studies have suggested that drug use significantly increases the risk of infection by SARS-CoV-2^6^ and leads to worse prognosis, particularly use of drugs that affect lung functions.^7-9^ Previous evidence has shown that cannabis and tobacco users are at higher risk of infection by influenza and of developing severe respiratory syndromes. Moreover, studies with animal models have shown that smoked drugs cause changes to the respiratory system from the nasal mucosa to the lung parenchyma.^10-12^ It is also necessary to consider that people who use cannabis and crack cocaine may share drugs or pipes with other people, and that concomitant use of tobacco may further increase risks.^13-17^ Considering these clinical findings, it might be the case that smoked drugs increase the risk of contamination by SARS-CoV-2. However, studies assessing the relationship between smoked drug consumption and SARS-CoV-2 are still scarce and have not received enough attention in global health recommendations.^18^

In Brazil, prevalence rates of regular use are 9.8% for tobacco^19^ and 2.5% for marijuana.^20^ Although these rates are lower than in many other countries, populations with low socioeconomic status have higher rates of use than the general population.^21,22^ Additionally, Brazil is one of the largest crack cocaine markets^23^ worldwide and 0.9% of the population use crack.^20^ Recent studies have shown that crack cocaine users are subject to multiple clinical and social vulnerabilities^24^ and that the prevalence of diseases associated with the severity of COVID-19 may be higher among users of tobacco, marijuana, and crack cocaine than in the general population.^6^

In the context of the current pandemic, in which social distancing is one of the most important strategies to mitigate the spread of SARS-CoV-2, there has been an increase in consumption of psychoactive drugs, which can make the respiratory and cardiovascular systems more susceptible to inflammatory complications caused by the new coronavirus. The reasons for this are not yet entirely clear, but it is speculated that stress caused by social isolation increases the likelihood of psychoactive drug use as a strategy to regulate anxiety, irritability, and sadness.^18^ A web-based survey with 11,391 participants that began eight days after official social distance rules were issued by the government, reported an increase of 31.2% in cannabis use, as well an increase of 35.6% in tobacco use, and 24.8% in alcohol use. This study suggests that the early phase of COVID-19 containment led to widespread increases in addiction-related behavior in the general population as a consequence of decreased well-being and increased stress.^25^

Currently, Brazil is one of the global epicenters of the pandemic,^26,27^ and vulnerable Brazilian populations are among the most severely affected in the world, constituting an emerging public health issue. Nevertheless, research addressing the consequences of drug use for progression of the pandemic is still scarce. This paper aims to briefly review the relationship between use of smoked drugs and COVID-19.

## Tobacco

Smoking is known to increase the risk for several comorbidities, including diabetes,^28^ cardiovascular disease,^29^ and chronic obstructive pulmonary disease (COPD),^30^ all of which appear to play an important role in the pathology of COVID-19 in humans.^31-33^ Furthermore, smoking increases expression of Angiotensin-converting enzyme 2 (ACE2), a receptor with high affinity for SARS-CoV-2 binding.^34,35^ Previous data suggest that tobacco influences the risk of transmission and severity of COVID-19. Results in the current literature on the severity of COVID-19 in the smoking population are still controversial.^36^ Nonetheless, a recent review has reported that smokers and ex-smokers show increased expression of ACE2 (the receptor for SARS-CoV-2), which may make these risk groups more susceptible to SARS-CoV-2 infection by different routes compared to non-smokers.^37^ A recent meta-analysis including 19 primary studies, comprising a pooled sample of 11,590 COVID-19 patients, suggested that smokers (both current and former) showed 1.91 times the odds of progression of COVID-19 compared to people who have never been smokers.^38^ Corroborating these findings, previous studies showed that tobacco is an important risk factor for hospitalization by and severity of similar diseases, such as MERS^39^ and H1N1.^40,41^

Conversely, other studies have suggested that the nicotinic acetylcholine receptor plays a key role in the pathophysiology of COVID-19 and may constitute a prevention strategy.^42^ However, the data supporting the hypothesis that nicotine helps to prevent COVID-19 are still scarce, and the results remain controversial.^43,44^

## Electronic cigarettes or vaporizers, and hookahs

Tobacco is available in different forms for consumption, with electronic cigarettes – or vaporizers – and hookahs becoming popular among young people.^45^ Electronic cigarettes, or e-cigarettes, deliver nicotine mixed with several types of flavors, making “vaping” (inhaling vapor created by e-cigarettes) a more palatable experience than smoking. Electronic cigarettes and hookahs are currently a social experience for young people because they may be shared in groups, which increases the risks of viral transmission.^45,46^ These devices have affordable prices and convey a false impression of being less harmful than smoking.^47^ In reality, they may be even more damaging than industrialized cigarettes, since a hookah session of 20 to 80 minutes is equivalent to exposure to 100 cigarettes.^48^ The concentration of nicotine in these devices is unknown due to an absence of regulatory policies and they may therefore be more toxic than traditional cigarettes.

At the end of 2019, the United States Center for Disease Control and Prevention (CDC) warned about the consequences of pulmonary injuries due to use of e-cigarettes.^49^ Symptoms of e-cigarette and hookah abuse include breathing difficulties, chest pain, and gastrointestinal conditions. These symptoms are a consequence of the flavors employed in those devices, which cause damage to pulmonary alveoli, resulting in thickening and narrowing of airways and causing symptoms similar to those observed in chronic obstructive pulmonary disease.^50-52^ In addition, early-stage studies show that e-cigarettes increase the virulence and inflammatory potential of pathogens such as *Streptococcus pneumoniae* and have other deleterious biological effects.^53^

A systematic review including 238 cases showed that, for respiratory injuries resulting from e-cigarette use, the most common diagnosis was e-cigarette, or vaping, product use-associated lung injury (EVALI) syndrome,^54^ a condition that exhibits worse progression when associated with viral infections.^55-57^ The CDC defines EVALI as a lung injury disease with symptoms similar to those of pneumonia in e-cigarette users or vapers, including respiratory (coughing, shortness of breath, and chest pain), gastrointestinal (nausea, vomiting, and diarrhea), and non-specific (fatigue, fever, and weight loss) symptoms. Diagnosis of EVALI can be difficult due to the similarity of its symptoms to those of other respiratory illnesses.^58^ The overlap of symptomatology and laboratory findings make diagnosis of EVALI challenging, particularly during the COVID-19 pandemic.^59^

There are currently no conclusive studies showing an association between EVALI and COVID-19. Nevertheless, we can identify similarities between the symptoms of these two diseases. Recent research has shown that use of e-cigarettes is a significant risk factor for COVID-19. Electronic cigarette users are five times more likely to be diagnosed with COVID-19. Dual users (those who smoke both traditional and electronic cigarettes) are seven times more likely to test positive for the virus. This may be explained by the effects of traditional cigarettes and e-cigarettes on the respiratory and immune systems.^47^

## Cannabis

Some studies have highlighted the increased sales of cannabis-related products during the COVID-19 pandemic.^60-62^ A Canadian online survey with teenagers aged 16-18 years (n = 1,054) showed an increase in use of cannabis and its derivatives around 3 weeks after social distancing began.^63^ The Global Drug Survey special edition on COVID-19 reported a 17.2% increase in smoked marijuana use and a 14.7% increase in use of products derived from marijuana in Brazil during the first months of the pandemic.^64^

It is still unclear whether cannabis use leads to the same COVID-19 outcomes observed in tobacco smokers. However, a study has reported evidence that marijuana users may be at increased risk if infected with coronavirus.^9^ It is well known that cannabinoids have deleterious effects on pulmonary function and inflammation by affecting the release of respiratory pathogens, which might increase the “cytokine storm” in COVID-19.^65^ Long-term cannabis consumption leads to effects similar to those observed in tobacco users or even more clinically deleterious effects.^66,67^

A recent study reported the case of a patient with a history of asthma who sought care at an emergency room with symptoms resembling those of COVID-19: fever, chills, dry cough, dyspnea, myalgia, increased respiratory rate, diarrhea, and low saturation. A chest X-ray showed bilateral interstitial infiltrates. As a result of clinical suspicion, the patient was isolated and treated for SARS-CoV-2, which was later ruled out by testing. After a positive toxicological result for marijuana, the patient acknowledged using the substance with e-cigarettes 3 times per week for the past two years. This prompted a change in the therapeutic approach. Despite being a case report, this study highlights the importance of differential diagnosis.^68^

## Crack cocaine

Another smoked drug that warrants concern is crack cocaine. Brazil is one of the main crack-consuming markets in the world and this drug is used mainly by vulnerable populations.^23^ Government and health professionals should dedicate special attention to crack users, since crack is the fifth leading cause of addiction worldwide and is associated with clinical and psychiatric comorbidities. Of illicit drugs, crack cocaine is one of the leading causes of seeking care from the health system.^69,70^ Crack use is strongly associated with negative health outcomes, such as HIV/AIDS, hepatitis, tuberculosis, and respiratory problems.^15,17,24^ Moreover, use of other substances, such as alcohol, tobacco, and marijuana, is common among crack-users.^71^ A recent study found that 84% of crack users hospitalized for withdrawal also consumed tobacco.^17,72^ It should be noted that the behavioral pattern of crack use is quite distinct from that of other smoked drugs such as tobacco and cannabis.^73^ Additionally, crack use usually occurs in unhealthy environments with no social distancing, which increases the risk of contamination by SARS-CoV-2.^74^

Crack smoke may cause acute pulmonary syndrome and pulmonary vasoconstriction, leading to ischemic cell damage with symptoms such as coughing, shortness of breath, and severe chest pain.^75^ Such symptoms may be easily confused with those of COVID-19, or they may increase the severity of COVID-19 respiratory symptoms. It is worth mentioning that the social stigma associated with crack users can hinder their access to the health system, thus increasing mortality within this population, whether due to COVID-19 or other morbidities. One of these well-known comorbidities is metal poisoning, caused by use of metal cans as the main crack smoking device. Metal poisoning may have immunosuppressive effects, affecting cellular and humoral immunity.^76^ To our knowledge, there are currently no studies with individuals who use crack and are infected by SARS-CoV-2, but we expect such data to be published eventually, as the clinical and psychological profiles of these subjects makes them more susceptible to contagion and worsening of COVID-19. It is important to emphasize that, in addition to physical risks of contamination and to worse prognosis for COVID-19, mental health problems are also common among crack users. Thus, stress resulting from the pandemic may be a trigger for relapse and increased consumption.^24^

## Conclusion

Use of smoked drugs, particularly tobacco, is associated with lung diseases that are risk factors for contamination by SARS-CoV-2. Additionally, in much of the West, drug use has increased since social distancing began in response to the COVID-19 pandemic; such an increase may contribute to aggravating the effects of the pandemic through the consequences of smoking and drug sharing for the health of users. It is essential to develop strategies based on specific characteristics of drug users and for mental health professionals to be part of strategic teams. It is also necessary to invest in information campaigns regarding risks and prevention of harm caused by smoked drugs, as well as to design strategies that facilitate access to psychosocial treatment during the pandemic.
